# Claim of Priority in the Reformation of the Treatment of the Insane
*"De la Réforme de Traitement des Aliénés." Par Dr. C. Pinel, Neveu. pp. 16. Paris: 1855.


**Published:** 1855-10-01

**Authors:** 


### CLAIM OF PRIORITY IN THE REFORMATION OF THE TREATMENT OF THE INSANE.*

In the last January number of this Journal, p. 72, we had occasion to refer to our previously strongly expressed conviction that to Pinel is due all the glory of having transformed the abode of the lunatic from a dungeon to a drawing-room or library. (January, 1854, p. 152.) We then alluded to our sentiments upon this well known fact in the history of Psychological Science, in consequence of a counter-claim recently advanced by M. Brierre de Boismont, on behalf of a comparatively obscure psychologist, M. Daquin, of Chambery. To that claim we gave the respect and attention to which we deemed it entitled from the high reputation of its advocate. A descendant, however, of the great Pinel has come forward to challenge the statements of M. Brierre de Boismont. Dr. C. Pinel vindicates the title of his illustrious ancestor to the exalted and glorious position hitherto accorded to him by his contemporaries and by posterity.

Justice to the memory of Pinel, and to the filial vindication thereof by his

* "De la Réforme de Traitement des Aliénés." Par Dr. C. Pinel, Neveu. pp. 16. Paris: 1855.

nephew, as well as the consideration of the great importance of this question in the history of psychological medicine, require that we should fully give the refutation of a statement to which we had, it seems, lent some weight by the space we devoted thereto on the occasion above-mentioned. Daquin, says M. Brierre de Boismont, published his "Medico-Philosophical Treatise upon Insanity" at Chambery in 1791, and at Paris in 1792. Pinel's treatise, having a similar title, was published in 1801. The interval of ten years was surely enough to have established the claim to priority, it may plausibly be urged.

Two essential elements of the controversy have, however, to be borne in mind in determining the dispute. First, the matter of fact: second, the nature of the views propounded by the respective authors. By the help of Dr. C. Pinel we shall enlighten our readers on these two primary branches of the inquiry.

1. Pinel, in 1783, was in charge of the inmates of the *Maison de Santé* of Dr. Belhomme, where he had already, on a small scale, put in practice those reforms which he subsequently carried out more extensively in the Bicêtre; he was at the same time chief editor of the *Gazette de Santé*, in which he had inserted numerous articles upon the treatment of the insane; in 1789, he published therein an article more especially inculcating the moral treatment of insanity. Before the first revolution, he had communicated to the *Société Royale de Médecine* an essay on the classification and moral treatment of lunatics; in 1790, Pinel successfully competed for a prize offered by the same learned body for an essay upon similar subjects; in 1790, he also inserted in the *Journal Gratuit de Santé* the history of a case of erotic melancholia cured by gardening, bathing, &c.; in 1791, he published in the journal, *La Médecine éclairée par les Sciences Physiques*, observations upon suicidal melancholy and its treatment by moral means. Towards the end of 1792, Pinel's celebrity in the department of psychological medicine obtained for him the appointment of Chief Physician to the Bicêtre, where one of his first acts was to liberate fifty of its manacled inmates. Verily this was the *fait accompli!* in the very same year that Daquin was publishing his ideas in a book that has scarcely survived its author, who would himself, probably, be amazed at the weight of honour now sought to be thrust upon him.

So much, then, for the matter-of-fact—the chronology of the writings of Daquin, and the writings and doings of Pinel.

2. The second division of our inquiry need not occupy us very long. We have seen that Pinel practically carried out ideas to which he laid not the claim of originality, but which he traced in the works of preceding authors as far back as Celsus and Cœlius Aurelianus. Daquin pretended to originality of the same ideas which he did not carry out, even on the small scale within his reach. M. Daquin's treatise, so far, then, in its advocacy of the moral treatment of insanity, differed not greatly from most of its predecessors. Pinel showed that chains could be dispensed with. Daquin was silent on the matter of chains. Pinel, liberal and just, did not hesitate or fear to direct attention to all writers of works possessing sufficient merit to have attracted his notice; his silence, therefore, with regard to Daquin's work, which is dwelt upon by M. Brierre as evidence of disingenuous plagiarism on the part of Pinel, tells rather in another direction, viz., as his nephew infers, that Daquin's work was among those of which he "*pris le parti sage de les passer sous silence.*" Pinel was too nobly engaged in the practical enforcement of his enlightened views to have condescended to such petty jealousy as is here insinuated.

We have thus, following Dr. C. Pinel's defence, discussed the essential elements of the controversy, and have, we trust, done strict justice to the memory of the great Pinel.

